# The use of a Mamdani-type fuzzy model for assessing the performance of a boom stabilization systems in a field sprayer

**DOI:** 10.1038/s41598-023-46087-y

**Published:** 2023-10-30

**Authors:** Zdzisław Kaliniewicz, Piotr Szczyglak, Adam Lipiński, Piotr Markowski, Seweryn Lipiński

**Affiliations:** Faculty of Technical Sciences, University of Warmia and Mazury in Olsztyn, 10-957 Olsztyn, Poland

**Keywords:** Plant ecology, Mechanical engineering, Scientific data

## Abstract

Fuzzy logic models are increasingly used to control simple and complex devices, as well as entire operating systems. In this study, a fuzzy logic model was applied to assess the performance a boom stabilization system in a field sprayer. The model was tested on a field sprayer with a trapezoid system for stabilizing the sprayer boom with a length of 21 m. Measuring cables for registering the displacement of the boom's terminal segments (right and left) in the vertical and horizontal plane were installed on the sprayer. The field sprayer was connected to a tractor. The model was based on two linguistic variables: "absolute displacement of the boom's terminal segments" and "boom stability index". It was assumed that the sprayer boom was stable when the displacement of the boom's terminal segments did not exceed 0.25% of boom length. The study demonstrated that the proposed model can be reliably used to assess boom stability in real time (during field operations). The time required to achieve boom stability was more than 2.5 times shorter in the vertical than in the horizontal plane, which can be attributed mainly to the structure of the stabilization system. The proposed model is universal, and it can be applied to evaluate other boom stabilization systems in field sprayers.

## Introduction

Ambiguous and imprecise objects, phenomena, and processes can be formally described with the use of the fuzzy set theory^1–3^. This is because real-world phenomena are much easier to describe with qualitative than quantitative variables. Fuzzy qualitative concepts such as a "tall" man, a "young" woman, or "low" temperature represent a certain range of contextual values, and they can be differently interpreted. This theory was put into practice by Mamdani^4,5^ who proposed a fuzzy inference control system. Numerous Mamdani-type fuzzy controllers have been developed over the years, and they are widely used in technical sciences and engineering. A fuzzy control system is based on a set of fuzzy logic rules, and it relies on precise measurements or ambiguous linguistic variables from an expert knowledge base^1–3,6,7^.

**Figure 1:**
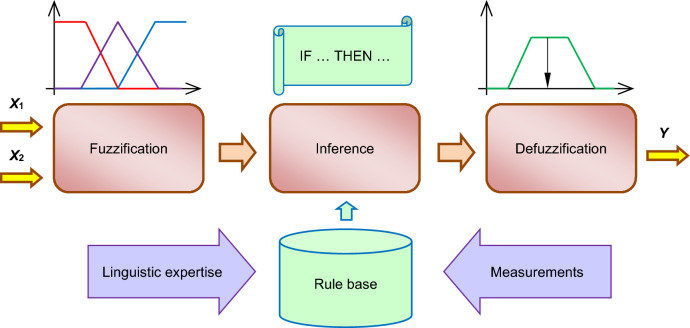
Fuzzy control diagram^5^.

The main advantage of fuzzy models over conventional mathematical models is that they require far less information about the modeled system. The collected data do not have to be precise or certain. Fuzzy systems convert quantitative input values into linguistic variables, use a set of rules to describe the analyzed phenomena, and convert fuzzy output signals into quantitative variables. A typical fuzzy model consists of three units: fuzzification, inference, and defuzzification^1–3,7,8^ (Fig. 1). Inference calculations are conducted with the use of a set of logic rules that are created based on expert knowledge (linguistic modeling) or analytical data from measurement systems or mathematical models. The results are used to determine cause and effect relationships between fuzzy sets of input and output values^1–3,6,7,9,10^.

Fuzzy controllers are widely used in various systems, objects, machines, and devices, including household equipment, autonomous vehicles, and complex operating systems^3,8,11–15^. Fuzzy controllers are also applied in agriculture^3^, mainly to control agricultural robots^16,17^, but also to manage greenhouses^18^, sort fruit and vegetables^19–21^, monitor soil parameters^22^, control irrigation systems^23–25^, and provide decision-making support in planning and performing farming operations^26–31^.

The application of crop protection chemicals is one of the most important treatments in intensive agriculture that is usually performed with field sprayers. This operation should involve technically sound machinery to ensure that the applied chemicals are uniformly distributed over the field^32,33^. According to Reynaldo et al.^34^ and Kappaun et al.^35^, effective delivery of crop chemicals is influenced not only by the type of field sprayer, but also by the structure and position of the sprayer boom, as well as the manner in which the boom is attached to the sprayer frame. During field operations, the displacement of all boom segments relative to the field should be minimal (the boom should be positioned at a height of around 0.5 m), and displacement in the horizontal plane should also be controlled. For this reason, modern agricultural sprayers are equipped with various boom stabilization systems to minimize displacement when the tractor moves on uneven terrain and to restore the boom to its original, neutral position^36^.

**Figure 2:**
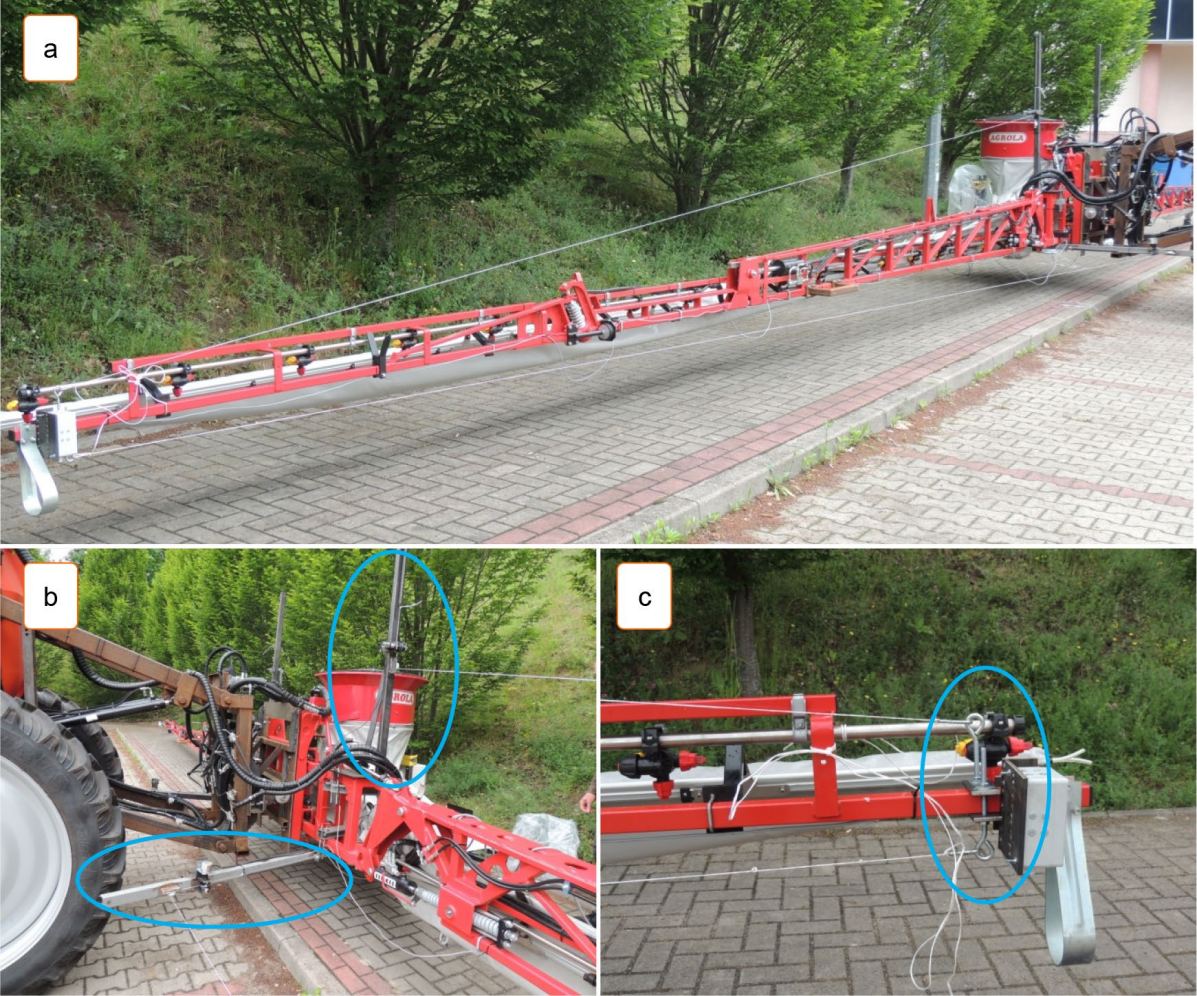
System for measuring the sprayer boom's displacement from a state of equilibrium: (**a**) general view; (**b**) support arms with reel spools and cable tension springs; (**c**) loop screws for attaching measuring cables.

**Figure 3:**
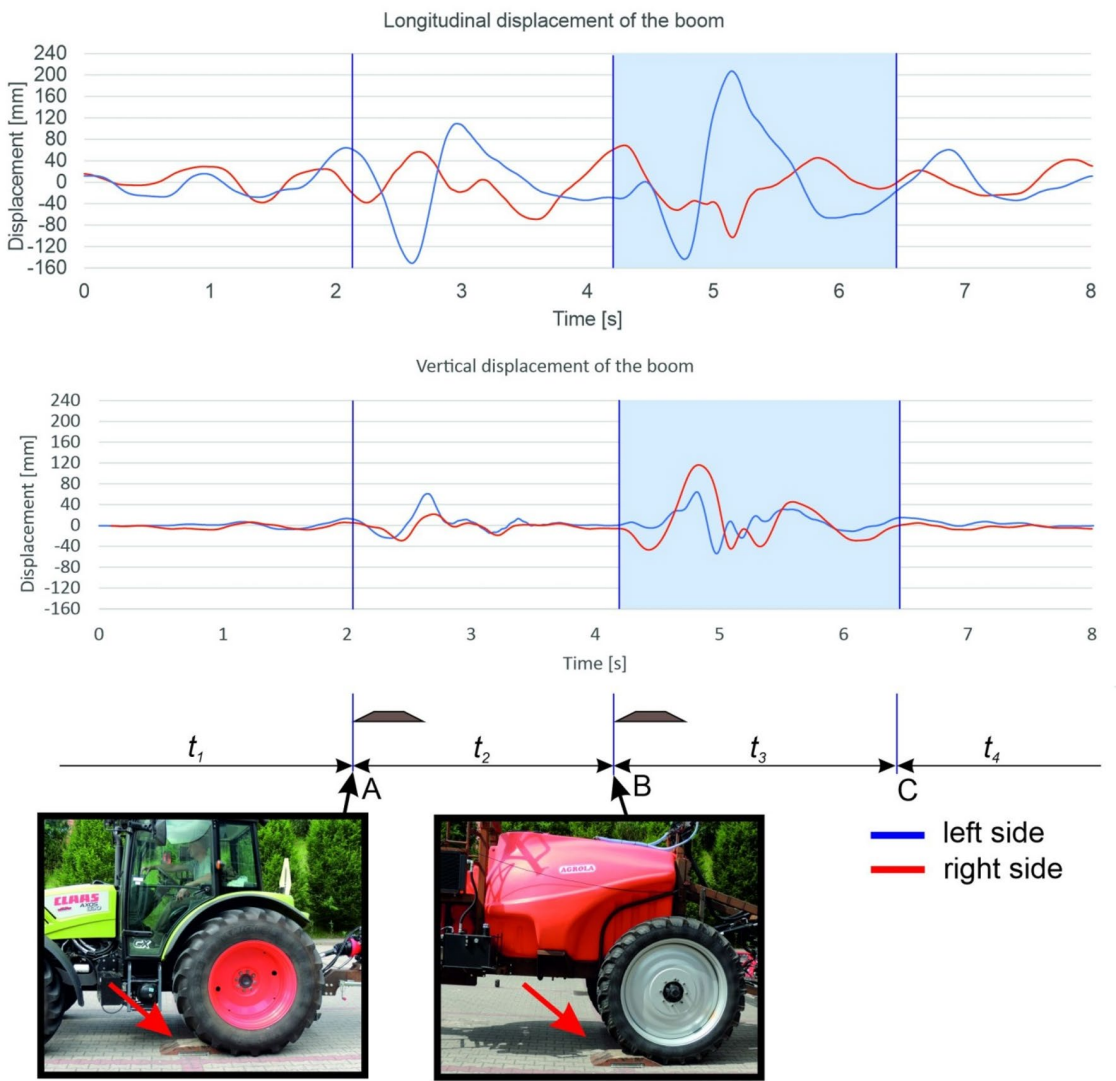
Displacement of the boom's terminal segments in two planes (speed – 8 km h^−1^, without an air sleeve): A—point of contact between the tractor’s rear wheel and the obstacle, B—point of contact between the sprayer unit’s wheel and the obstacle, C—point at which the sprayer boom was stabilized, *t*_1_—time directly before the tractor's rear wheel came into contact with the obstacle, *t*_2_—time interval between points at which the tractor wheel and the sprayer wheel came into contact with the obstacle, *t*_3_—time of unstable boom operation after the sprayer wheel came into contact with the obstacle, *t*_4_—time of stable boom operation.

The sprayer boom is controlled with the use of specialist devices that register the movement of all boom segments^33,34,37^. These measurements can be conducted in laboratory track simulators or in the field. Changes in boom position are registered by cameras or sensors^35,38–42^. A relatively simple method for monitoring boom displacement from a neutral position was proposed by Kaliniewicz et al.^43^. The cited authors developed a system of four measuring cables (to calculate the threshold displacement of all boom segments in the horizontal and the vertical plane) that are wound on the spools of measuring reels and are connected to sensors that measure the reel's angle of rotation (Fig. 2). Each reel's angle of rotation is measured by an encoder connected to a rapid data acquisition system, and signals are registered with the same sampling frequency of 1 kHz. The proposed system can also be used to measure the speed and acceleration of the boom's terminal segments in both planes, and to determine the resultant values of the examined parameters. To minimize the noise caused by cable vibration, the moving average is calculated from 10 successive measuring points. The registered data can be plotted to visualize the displacement of the boom's terminal segments (Fig. 3). However, the periods when the boom remains in a stable position and the time needed to restore the boom's stability cannot be clearly inferred from the results. Therefore, the aim of the present study was to develop a fuzzy rule-based model for identifying the stability states of a field sprayer boom.

## Model for identifying the stable operation of a sprayer boom

The model for identifying a sprayer boom's stability states was developed based on a Mamdani-type fuzzy inference system. The linguistic variable "absolute displacement of the boom's terminal segments" ǀ*d*_*l*_ǀ (right and left arm) was analyzed in the horizontal and vertical plane. Three linguistic spaces were assigned to the above variable:

- Admissible displacement,
- Threshold displacement,
- Excessive displacement.

**Figure 4:**
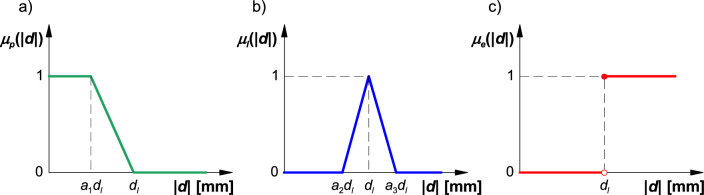
Membership functions: (**a**) admissible displacement, (**b**) threshold displacement, (**c**) excessive displacement; *a*_1_, *a*_2_, *a*_3_—coefficients, *d*_*l*_—threshold displacement.

The membership function of admissible displacement of the boom's terminal segment was described with an Z-curve (external left) presented in Fig. 4a. This function can be described with the following equation:

1 $${\mu }_{p}\left(\left|d\right|\right)=\left\{\begin{array}{c}1\leftrightarrow \left|d\right|<{a}_{1}{\cdot d}_{l}\\ \frac{1}{1-{a}_{1}}\left(1-\frac{\left|d\right|}{{d}_{l}}\right)\leftrightarrow \left|d\right|\ge {a}_{1}{\cdot d}_{l}\cap \left|d\right|\le {d}_{l}\\ 0\leftrightarrow \left|d\right|>{d}_{l}\end{array}\right.,$$

where: *a*_1_ – coefficient (0 < *a*_1_ < 1), |*d*| – absolute displacement of the boom's terminal segment [m], *d*_*l*_ – threshold displacement [m].

The membership function of threshold displacement of the boom's terminal segment was described with a t-curve (triangular) presented in Fig. 4b. This function can be described with the following equation:

2 $${\mu }_{l}\left(\left|d\right|\right)=\left\{\begin{array}{c}0\leftrightarrow \left|d\right|\le {a}_{2}{\cdot d}_{l}\cup \left|d\right|\ge {a}_{3}{\cdot d}_{l}\\ \frac{\left|d\right|-{a}_{2}\cdot {d}_{l}}{{d}_{l}\cdot \left(1-{a}_{2}\right)}\leftrightarrow \left|d\right|>{a}_{2}{\cdot d}_{l}\cap \left|d\right|\le {d}_{l}\\ \frac{{a}_{3}\cdot {d}_{l}-\left|d\right|}{{d}_{l}\cdot \left({a}_{3}-1\right)}\leftrightarrow \left|d\right|>{d}_{l}\cap \left|d\right|<{{a}_{3}\cdot d}_{l}\end{array}\right.,$$

where: *a*_2_, *a*_3_ – coefficients (0 < *a*_2_ < 1; 1 < *a*_3_ < 2).

The membership function of excessive displacement of the boom's terminal segment was described with a curve presented in Fig. 4c. This function can be described with the following equation:

3 $${\mu }_{e}\left(\left|d\right|\right)=\left\{\begin{array}{c}0\leftrightarrow \left|d\right|\le {d}_{l}\\ 1\leftrightarrow \left|d\right|>{d}_{l}\end{array}\right..$$

The linguistic variable "boom stability index" *S* was applied to identify the boom's stability states. Three linguistic spaces were assigned to the above variable:

- Stable boom,
- Threshold boom stability,
- Unstable boom.

**Figure 5:**
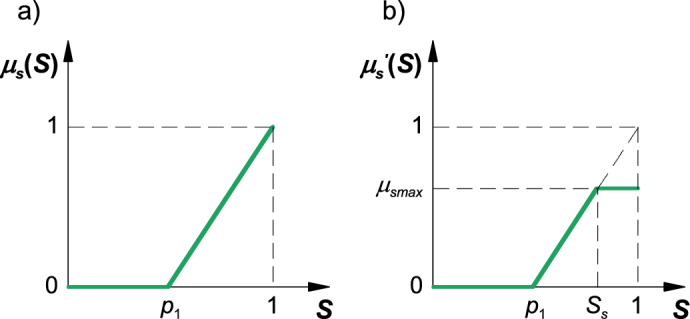
Membership function of the stable boom state (**a**) and the result function (**b**): *p*_1_—parameter, *S*_*s*_—coordinate on the x-axis, *μ*_*smax*_—coordinate on the y-axis.

The membership function of a stable boom state is presented in Fig. 5a. This function can be described with the following equation:

4 $${\mu }_{s}\left(S\right)=\left\{\begin{array}{c}0\leftrightarrow S\le {p}_{1}\\ \frac{S-{p}_{1}}{1-{p}_{1}}\leftrightarrow S>{p}_{1}\end{array}\right.,$$

where: *p*_1_ – function parameter (0 < *p*_1_ < 1), *S* – boom stability index.

A fuzzy implication of the stable boom state can be defined with the use of an equation for calculating the bounding coordinate on the y-axis based on the registered displacement of the boom's terminal segments (left and right) at a given moment:

5 $${\mu }_{smax}=\mathrm{min} \left\{{\mu }_{pl}\left(\left|d\right|\right), {\mu }_{pr}\left(\left|d\right|\right)\right\},$$

where: *μ*_*pl*_(|*d*|) – value calculated from the membership function of admissible displacement of the boom's left terminal segment, *μ*_*pr*_(|*d*|) – value calculated from the membership function of admissible displacement of the boom's right terminal segment.

The resulting function is presented in Fig. 5b, and it can be described with the following equation:

6 $${\mu }_{s}{\prime}\left(S\right)=\left\{\begin{array}{c}0\leftrightarrow S\le {p}_{1}\\ \frac{S-{p}_{1}}{1-{p}_{1}}\leftrightarrow S>{p}_{1}\cap S<{S}_{s}\\ {\mu }_{smax}\leftrightarrow S\ge {S}_{s}\end{array}\right.,$$

where: *S*_*s*_ – coordinate on the x-axis, *μ*_*smax*_ – bounding coordinate on the y-axis.

The coordinate on the x-axis can be calculated with the use of the following equation:

7 $${S}_{s}={\mu }_{smax}\cdot \left(1-{p}_{1}\right)+{p}_{1}.$$

**Figure 6:**
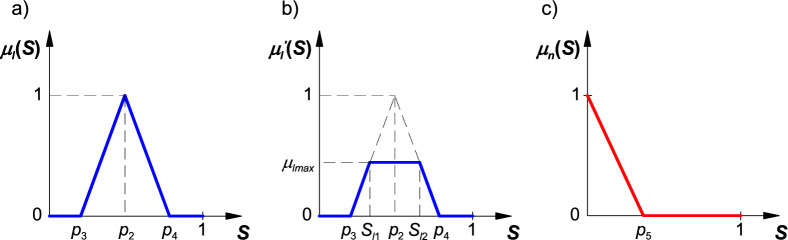
Membership function of threshold boom displacement (**a**), the result function (**b**), and the membership function of unstable boom state: *p*_2_…* p*_5_—coefficients; *S*_*l*1_, *S*_*l*2_—coordinates on the x-axis; *μ*_*lmax*_—bounding coordinate on the y-axis.

The membership function of threshold boom stability is presented in Fig. 6a, and it is described with the following equation:

8 $${\mu }_{l}\left(S\right)=\left\{\begin{array}{c}0\leftrightarrow S\le {p}_{3}\cup S\ge {p}_{4}\\ \frac{S-{p}_{3}}{{p}_{2}-{p}_{3}}\leftrightarrow S>{p}_{3}\cap S\le {p}_{2}\\ \frac{{p}_{4}-S}{{p}_{4}-{p}_{2}}\leftrightarrow S>{p}_{2}\cap S<{p}_{4}\end{array}\right.,$$

where: *p*_2_ – function parameter (*p*_3_ < *p*_2_ < *p*_4_), *p*_3_ – function parameter (0 < *p*_3_ < *p*_2_), *p*_4_ – function parameter (*p*_2_ < *p*_5_ < 1).

A fuzzy implication of threshold boom stability was defined with the use of an equation for calculating the bounding coordinate on the y-axis based on the product of registered displacements (absolute values) of the boom's terminal segments (left and right) at a given moment:

9 $${\mu }_{lmax}={\mu }_{ll}\left(\left|d\right|\right)\cdot {\mu }_{lr}\left(\left|d\right|\right),$$

where: *μ*_*ll*_(|*d*|) – value calculated from the membership function of threshold displacement of the boom's left terminal segment, *μ*_*lr*_(|*d*|) – value calculated from the membership function of threshold displacement of the boom's right terminal segment.

The resulting function is presented in Fig. 6b, and it can be described with the following equation:

10 $${\mu }_{l}{\prime}\left(S\right)=\left\{\begin{array}{c}0\leftrightarrow S\le {p}_{3}\cup S\ge {p}_{4}\\ \frac{S-{p}_{3}}{{p}_{2}-{p}_{3}}\leftrightarrow S>{p}_{3}\cap S<{S}_{l1}\\ {\mu }_{lmax}\leftrightarrow S\ge {S}_{l1}\cap S\le {S}_{l2}\\ \frac{{p}_{4}-S}{{p}_{4}-{p}_{2}}\leftrightarrow S>{S}_{l2}\cap S<{p}_{4}\end{array}\right.,$$

where: *S*_*l*1_, *S*_*l*2_ – coordinates on the x-axis, *μ*_*lmax*_ – bounding coordinate on the y-axis.

The coordinates on the x-axis can be calculated with the use of the following equation:

11 $${S}_{l1}={\mu }_{lmax}\cdot \left({p}_{2}-{p}_{3}\right)+{p}_{3},$$

12 $${S}_{l2}={p}_{4}-{\mu }_{lmax}\cdot \left({p}_{4}-{p}_{2}\right).$$

The membership function of the unstable boom state is presented in Fig. 6c, and it is described with the following equation:

13 $${\mu }_{n}\left(S\right)=\left\{\begin{array}{c}1-\frac{S}{{p}_{5}}\leftrightarrow S<{p}_{5}\\ 0\leftrightarrow S\ge {p}_{5}\end{array}\right.,$$

where: *p*_5_ – function parameter (0 < *p*_5_ < 1).

A fuzzy implication of the unstable boom state can be defined with the use of an equation for calculating the bounding coordinate on the y-axis based on the registered displacement of the boom's terminal segments (left and right) at a given moment:

14 $${\mu }_{nmax}=\mathrm{max} \left\{{\mu }_{el}\left(\left|d\right|\right), {\mu }_{er}\left(\left|d\right|\right)\right\},$$

where: *μ*_*el*_(|*d*|) – value calculated from the membership function of excessive displacement of the boom's left terminal segment, *μ*_*er*_(|*d*|) – value calculated from the membership function of excessive displacement of the boom's right terminal segment.

The discussed coordinate can assume only two values for the adopted membership function of excessive boom displacement: 0 – when the displacement of the boom's each terminal segment does not exceed threshold displacement, or 1 – when the displacement of at least one terminal segment is equal to or exceeds threshold displacement.

**Figure 7:**
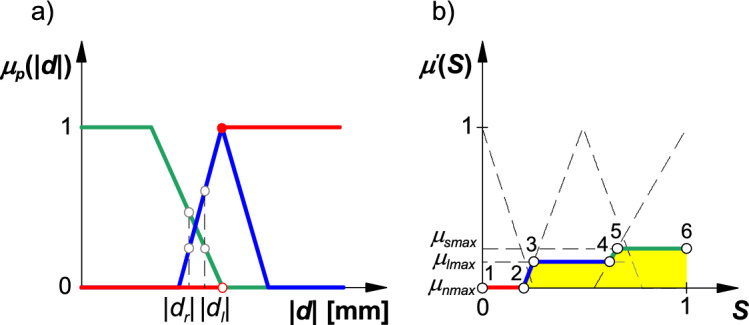
Membership functions of boom stability states (**a**) and the result of rule aggregation (**b**) for a field sprayer boom: |*d*_*l*_|, |*d*_*r*_|—absolute values of threshold displacement of the boom's left and right terminal segments; *μ*_*lmax*_, *μ*_*nmax*_, *μ*_*smax*_—bounding coordinates on the y-axis for: threshold displacement, unstable boom state, and stable boom state.

The results of rule aggregation are presented for a scenario where the displacement of the boom's left terminal segment is somewhat greater than the displacement of the right terminal segment, and both displacements do not exceed threshold displacement (Fig. 7). The bounding coordinate on the y-axis will assume the following values: 0—for excessive displacement; the product of the membership functions of threshold displacement of the boom's right and left terminal segments—for threshold displacement; the membership function of admissible displacement of the boom's left terminal segment—for admissible displacement. The final value of the boom stability index is calculated with the center of gravity method^2,9,44,45^ using the following formula:

15 $$S=\frac{\sum_{i=1}^{n}{S}_{i}\cdot {\mu }{\prime}\left({S}_{i}\right)}{\sum_{i=1}^{n}{\mu }{\prime}\left({S}_{i}\right)},$$

where: *n* – number of characteristic points on the rule aggregation curve (*n* = 6 in the analyzed scenario), *S*_*i*_ – coordinate of the *i*-th point on the x-axis, *μ’*(*S*_*i*_) – coordinate of the *i*-th point on the y-axis.

## Results and discussion

The ISO 14,131 standard^46^ does not include any coefficients for assessing the stability of a field sprayer's boom. Therefore, the parameters of the model for identifying boom stability were selected on the assumption that the distance between spray nozzles should not exceed 0.1 m or 0.5% of the boom's total length^47^. It was assumed that the threshold displacement of each terminal segment in the vertical plane should not exceed 0.25% of the boom's working width. This parameter was set at 52.5 mm for the tested boom with a length of 21 m. The same threshold displacement was used to determine the boom's stability in the horizontal plane. The parameters of all equations for calculating the boom's displacement and stability states are presented in Table 1.

**Table 1:** Parameters of the model for identifying the stability states of a field sprayer boom.

Parameter	Value
Threshold displacement [mm]	52.5
Coefficient *a*_1_ [ −]	0.5
Coefficient *a*_2_ [ −]	0.9
Coefficient *a*_3_ [ −]	1.1
Coefficient *p*_1_ [ −]	0.5
Coefficient *p*_2_ [ −]	0.5
Coefficient *p*_3_ [ −]	0.25
Coefficient *p*_4_ [ −]	0.75

**Figure 8:**
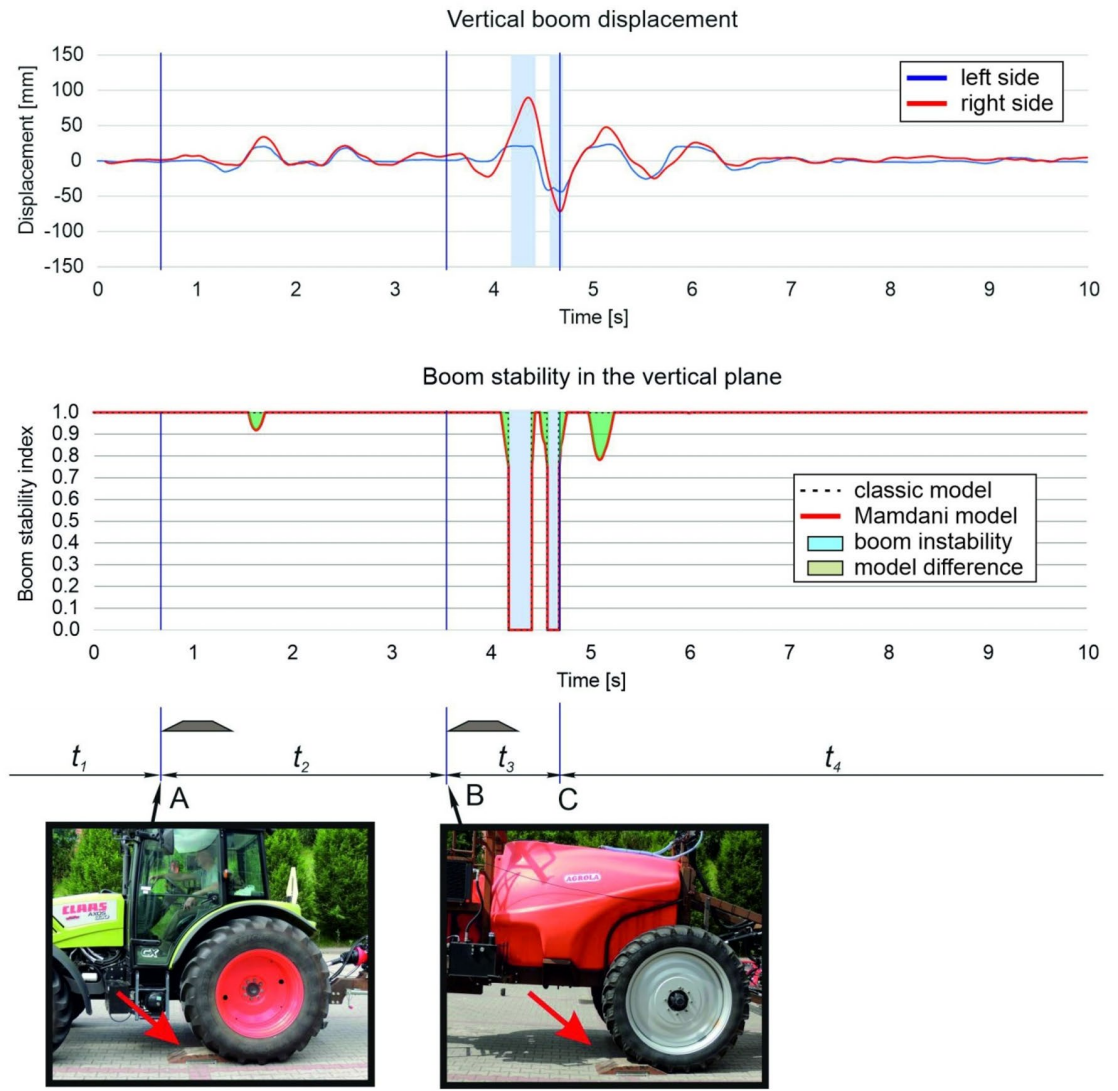
Time of boom displacement and boom stabilization in the vertical plane (speed—6 km h^−1^, without an air sleeve): *A*—point of contact between the tractor’s rear wheel and the obstacle, *B*—point of contact between the sprayer unit’s wheel and the obstacle, *C*—point at which the sprayer boom was stabilized, *t*_1_—time directly before the tractor's rear wheel came into contact with the obstacle, *t*_2_—time interval between points at which the tractor wheel and the sprayer wheel came into contact with the obstacle, *t*_3_—time of unstable boom operation after the sprayer wheel came into contact with the obstacle, *t*_4_—time of stable boom operation.

**Figure 9:**
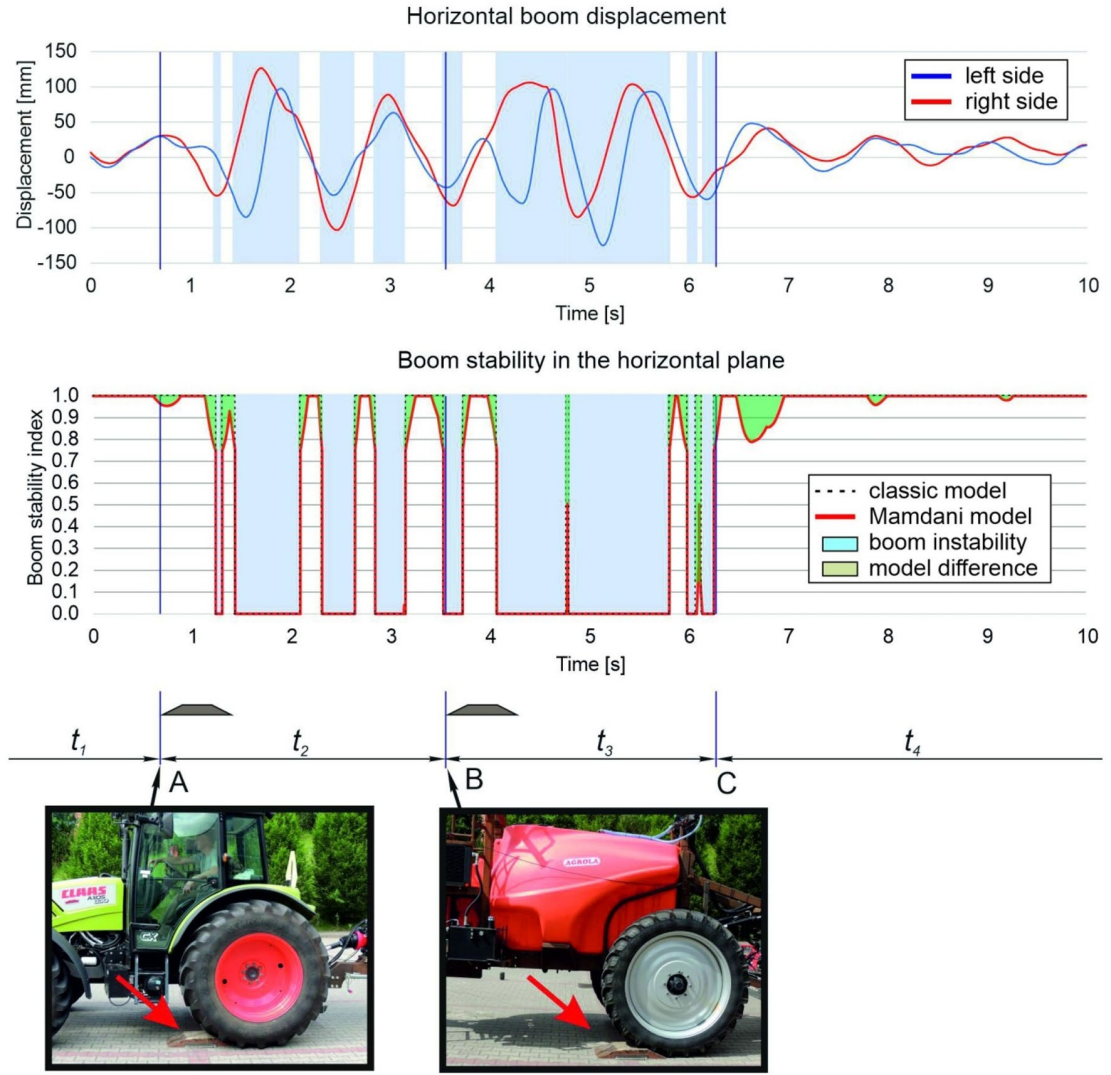
Time of boom displacement and boom stabilization in the horizontal plane (speed—6 km h^−1^, without an air sleeve): *A*—point of contact between the tractor’s rear wheel and the obstacle, *B*—point of contact between the sprayer unit’s wheel and the obstacle, *C*—point at which the sprayer boom was stabilized, *t*_1_—time directly before the tractor's rear wheel came into contact with the obstacle, *t*_2_—time interval between points at which the tractor wheel and the sprayer wheel came into contact with the obstacle, *t*_3_—time of unstable boom operation after the sprayer wheel came into contact with the obstacle, *t*_4_—time of stable boom operation.

The displacement of the boom's terminal segments in each stage of movement during a single test run are presented in Figs. 8 and 9. Similar values were reported by other authors^48^. At the tested speed, the displacement of the boom's left arm was somewhat smaller than the displacement of the right arm despite the fact that the obstacle was positioned on the left side of the sprayer unit. After the sprayer unit crossed the obstacle, boom displacement was greater in the horizontal than the vertical plane, probably because modern stabilization systems are designed to attenuate vibration only in the vertical plane^36,49,50^. However, according to many researchers^39,51,52^, boom displacement in the horizontal plane has a more adverse effect on spray quality than vertical displacement. When the boom is displaced from the sprayer frame in the horizontal plane, the dose applied to some plants could be insufficient, whereas other plants could be excessively sprayed with the chemical agent^53,54^.

Boom displacement was relatively small in the vertical plane, but significant in the horizontal plane when the rear wheel of sprayer unit came into contact with the obstacle. The peak-to-peak value of the greatest displacement in the boom's right terminal segment was estimated at 45 mm in the vertical plane and 230 mm in the horizontal plane. When the tractor crossed the obstacle, the trailer connector was lifted up and sideways, and a small rotation of the sprayer frame was observed, mainly in the vertical axis. Immediately after crossing the obstacle, the trailer connector returned to its initial position, and the sprayer boom was displaced once again. The horizontal displacement of the boom's terminal segments (not attenuated by the stabilization system) was considerable, and it was not reduced until the wheel of the sprayer unit came into contact with the obstacle. When the wheel came into contact with the obstacle, the left boom arm was lifted and rotated in the vertical and horizontal axis. This sudden movement generated high peak-to-peak values of threshold displacement. Right-side displacement was similar in both planes (approx. 160–180 mm), whereas left-side displacement was around 240% greater in the horizontal than in the vertical plane (approx. 20 mm and 65 mm, respectively). In both planes, terminal boom segments were not displaced far from the neutral position, but the return to the neutral position occurred more rapidly in the vertical than in the horizontal plane. Similar observations were made by Pochi and Vannucci^48^ who analyzed changes in the position of a spray boom with a working width of 12 m on uneven terrain. As previously mentioned, these differences can be attributed to the specificity of the stabilization system which reduces boom displacement mainly in the vertical plane, whereas displacement in the horizontal plane is only minimally attenuated^36,49,50^.

In the proposed model for identifying the stability states of a sprayer boom, instantaneous values of the boom stability index *S* were determined in both planes, and the duration of each stability state is presented in Figs. 8 and 9. Periods of unstable boom operation were also identified. These periods coincided with the moments when the displacement of at least one boom arm exceeded threshold values, which implies that the model correctly identified boom instability states. For comparison, Figs. 8 and 9 also present the values of the boom stability index in the conventional model which can be described with the following equation:

16 $$S=\left\{\begin{array}{c}0\leftrightarrow \left|d\right|>{d}_{l}\\ 1\leftrightarrow \left|d\right|\le {d}_{l}\end{array}\right..$$

This model can be used to identify two boom stability states. The boom is unstable when the stability index *S* equals 0, and it is stable when *S* equals 1. In the Mamdani model, the stability index can assume intermediate values in the range of 0 to 1 in a continuous manner. Threshold values of *S* (0 and 1) denote unstable and stable states, but the intermediate state is also possible. Therefore, the value of *S* describes the intermediate state relative to threshold states (stable or unstable boom). The differences between the models are marked in green in figure drawings. The stability index calculated in the Mamdani model has greater practical value because it can be used in boom stabilization systems as a parameter that predicts changes in boom position. The system's sensitivity can be modified by changing the parameters in the model.

When the wheel came into contact with the obstacle, the boom was destabilized only in the horizontal plane. Five short periods of boom instability were identified up to the moment when the wheel came into contact with the obstacle (time interval *t*_2_), and these periods accounted for around 48% of the analyzed time. After the sprayer unit crossed the obstacle, the stabilization system rapidly attenuated boom vibrations in the vertical plane, probably because both arms were propelled in the same direction during the maneuver. The boom was lifted when the wheels came into contact with the obstacle, after which it rapidly returned to the previous position near the boom's center of gravity. The chronometric analysis (Table 2) revealed that the average time required to stabilize the boom at the tested speed was 1.06 s. The threshold displacement of the boom's terminal segments (when *S* = 0) was exceeded in 30% of that time. Boom stabilization time was significantly longer (by approx. 170%) in the horizontal than in the vertical plane. The proportion of unstable operating time during that period also increased because the amplitude of displacement of both terminal segments relative to each other was shifted, and threshold displacement was exceeded for a longer period of time (not only for both arms, but also for the left and right arm separately). Boom operation was more stable in the vertical than in the horizontal plane directly before and after the rear wheel of the sprayer unit came into contact with the obstacle (sampling time of 10 s). During the entire test, the average time of unstable operation was determined at 0.23 s and 3.18 s, respectively, which indicates that the stabilization system significantly reduced boom displacement in the vertical plane. However, new solutions for stabilizing boom operation should be developed in the future to ensure that boom vibrations are effectively damped also in the horizontal plane.

**Table 2:** Chronometric analysis of the sprayer boom's stability states.

Parameter	Plane	
	Vertical	Horizontal
Registered time of travel [s]	10	
Time of stable operation *t*_*s*_ [s]	9.10 ± 0.08	3.92 ± 0.35
Time of semi-stable operation *t*_*h*_ [s]	0.67 ± 0.07	2.90 ± 0.49
Time of unstable operation *t*_*n*_ [s]	0.23 ± 0.08	3.18 ± 0.20
Stabilization time *t*_3_ [s]	1.06 ± 0.06	2.86 ± 0.25

## Materials and methods

**Figure 10:**
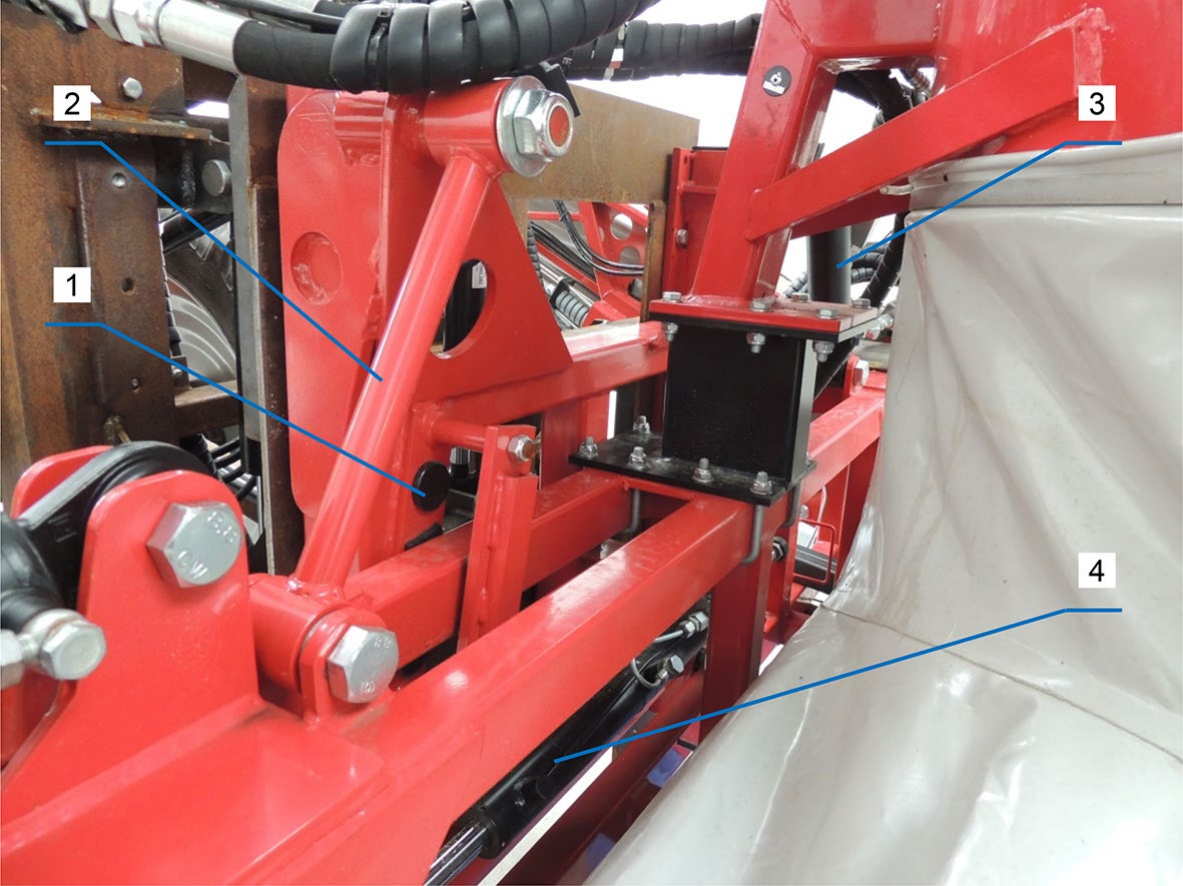
Stabilization system of a field sprayer boom: 1—slide bushings, 2—fixed-length bar, 3—hydraulic cylinder with adjustable length, 4—boom displacement damper in the vertical plane.

The proposed model was validated on a mobile test bench (field sprayer with an air sleeve, AGROLA Zdzisław Niegowski, Płatkownica, Poland) connected to a Claas AXOS 330 tractor (CLAAS Group, Harsewinkel, Germany). The sprayer was equipped with a 3 m^3^ tank and a hydraulic sprayer boom with a working width of 21 m. The boom was stabilized by a trapezoid suspension frame^30^ with a vertically mounted vibration damper (Fig. 10). The boom was mounted to the frame with two tension bars, including a hydraulic cylinder with adjustable length for changing the boom's position (in a direction parallel to the crop stand). Boom displacement in the vertical plane was controlled with the use of a hydraulic damper. The central segment of the sprayer boom is kept in equilibrium by the damper's sliding arms, which delays and attenuates boom responses to changes in the field sprayer's movement. The bottom bar in the stabilization system was fixed with two slide bushings (on the right and left side) to prevent excessive friction and to stabilize the central segment of the sprayer boom in the horizontal plane. The sprayer frame was set on two wheels (tire size 270/95R42).

A system for monitoring boom displacement from an equilibrium position was mounted on the field sprayer (Fig. 2). The monitoring system was described in detail by Kaliniewicz et al.^43^. The system consists of two identical units installed on both sides of the boom's central segment. Each unit comprises two supports for registering the displacement of the boom's terminal segments, one in the horizontal plane, and the other in the vertical plane. Displacement is measured with the use of cables. One end of the measuring cable is attached to the terminal segment of the sprayer boom, and the other end is wound onto one of the spools of the measuring reel connected to the motion sensor. One end of the tension cable is wound on the other spool, and the other end is attached to a tension spring. Constant tension is maintained on the measuring cable, and the cable is wound or unwound from the spool every time the terminal segment of the sprayer boom changes position. The reel's angle of rotation is converted to a digital signal by a rotation sensor. The system comprised four cables because the displacement of the boom's terminal segments was registered in two planes (horizontal and vertical) and on two sides of the field sprayer (left and right).

The boom stabilization system was tested and boom displacement was registered on 6 June 2022 according to ISO 14,131 guidelines^46^. A straight-line test track with an estimated length of 50 m was marked on a flat surface paved with concrete blocks. A wooden obstacle with the shape of an isosceles trapezoid in the vertical cross-section was installed on the test track along the path traveled by the sprayer unit's left wheels. Obstacle dimensions were described by Kaliniewicz et al.^43^. The obstacle was introduced to the test track to simulate real-world conditions and to set the sprayer boom into motion on uneven terrain. The sprayer tank was filled with water up to two-thirds of its volume (approx. 2 m^3^). Tire pressure was 0.35 ± 0.01 MPa, and wheel track width was 1.65 m for the tractor and the sprayer unit. The spray boom was positioned 0.7 m above the ground to enable a full range of motion during the test. The test was conducted in triplicate. Tractor speed was 6 km‧h^−1^, and the secondary blower system was not activated during the test because the inflatable sleeve could restrict the boom's movement. The test was conducted on a fair day at a temperature of around 22 ± 1 °C and wind speed of around 2 m‧s^−1^.

During the test, the sprayer unit moved along the test track, and its movement consisted of five successive stages: (1) acceleration to the preset speed, (2) movement at the preset speed, (3) crossing the obstacle, (4) movement at the preset speed along a distance of around 10 m, (5) braking. Data from motion sensors were registered to calculate the displacement of the boom's terminal segments in the horizontal and vertical plane with the use of the formulas described by Kaliniewicz et al.^43^. A moving average from 10 measuring points was calculated to minimize the impact of cable vibration on the displacement of the boom's terminal segments. The parameters of the model presented in Sect. “Model for identifying the stable operation of a sprayer boom” were chosen based on expert knowledge (one of the co-authors) to correlate the registered changes in boom position with each stage of the sprayer unit's movement along the test track. The following parameters were determined based on a chronometric analysis of the boom stability index *S*:

- Time of unstable boom operation *t*_*n*_,
- Time of stable boom operation *t*_*s*_,
- Time of semi-stable boom operation *t*_*h*_ (for 0 < *S* < 1),
- Time of boom stabilization *t*_3_ (from the moment the wheel came into contact with the obstacle to the moment when the displacement of the boom's terminal segments reached threshold displacement).

## Conclusions

The proposed fuzzy logic model can be applied to assess the operation of a field sprayer's boom and to identify the boom's stability states in real time. The model's parameters can be changed to modify the sensitivity of the measuring system. Subject to need, the model can be used to register the boom's stability states or to control the stabilization system.

The displacement of the boom's terminal segments is registered in the horizontal and the vertical plane, and the time during which the boom is stable, semi-stable, and unstable can be accurately determined based on the calculated values of the boom stability index. To simplify data readout, these values can be analyzed jointly to identify a common region of stable and unstable boom operation for both planes.

In the future, the developed model can be expanded to include the sprayer unit's speed and acceleration. The instantaneous values of these parameters can be registered together with boom displacement. It appears that the parameters describing the movement of the boom's terminal segment significantly influence the quality of spraying operations.

## Data Availability

The datasets generated during and/or analyzed during the current study are available from the corresponding author on reasonable request.
